# In Vitro Antioxidant and Anti-Colon Cancer Activities of *Sesamum indicum* L. Leaf Extract and Its Major Component, Pedaliin

**DOI:** 10.3390/foods10061216

**Published:** 2021-05-27

**Authors:** Seoyun Kim, Hyi Young Yang, Hwa Jin Lee, Jihyeung Ju

**Affiliations:** 1 Department of Food and Nutrition, Chungbuk National University, 1 Chungdae-ro, Cheongju 28644, Korea; star7073@daum.net (S.K.); robinyang@g.cbnu.ac.kr (HY.Y.); 2 School of Industrial Bio-Pharmaceutical Science, Semyung University, 65 Semyung-Ro, Jecheon, Chungbuk 27136, Korea; hwalee@semyung.ac.kr

**Keywords:** sesame leaf, pedaliin, oxidative stress, colon cancer

## Abstract

Sesame (*Sesamum indicum* L.) leaves (SLs) are used as vegetables and traditional medicines in Asian and African countries. We investigated in vitro antioxidant and anti-colon cancer efficacy of ethanol extract of SL (SLE) and its major bioactive component. SLE contained appreciable amount of major classes of antioxidant phytochemicals, such as total polyphenols, total flavonoids, and carotenoids, and correspondingly exhibited antioxidant activities, such as radical scavenging activity and ferric reducing antioxidant power (FRAP). A cell viability assay showed that SLE time- and dose-dependently attenuated the growth of human colon cancer cells, HT29 and HCT116. Flow cytometry analysis showed that SLE increased sub-G1 (in HT29 and HCT116) and G2/M (in HCT116) cell populations, suggesting that the growth inhibition by SLE was due to induction of apoptosis and G2/M cell cycle arrest. Trans-well and wound-healing assays showed that SLE alleviated invasion and migration of HT29 and HCT116 cells in non-cytotoxic conditions. High-performance liquid chromatography analysis revealed that pedaliin (6-hydroxylueolin 7-methyl ether 6-glucoside; pedalitin-6-*O*-glucoside) was a major constituent of SLE. Moreover, FRAP, growth-inhibitory, anti-invasive, and anti-migratory activities of pedaliin were found. These results demonstrated that SLE possesses in vitro antioxidant and anti-colon cancer activities and that pedaliin is a major component contributing to such activities.

## 1. Introduction

Sesame (*Sesamum indicum* L., Pedaliaceae family) is regarded as one of the oldest and most important oilseeds due to the high quantity and quality of oil in the seeds [1]. Sesame seeds and their constitutive lignans, such as sesamin and sesaminol, have been extensively studied, and their diverse biological activities have been well documented [2,3]. While the seed is the primary edible part in this plant, sesame leaves (SLs) are also used as vegetables and traditional medicines in different Asian and African countries [4,5]. Several studies have reported on the amino acid profile [5], phytochemical characterization [4,6,7,8,9], and biological activities of SLs [4,8,10,11]. In an amino acid profiling study, all essential amino acid were reported to be present in SLs [5]. In a phytochemical study, a new pentacyclic triterpene (3-epibartogenic acid), epigallocatechin, and a kaempferol glycoside were isolated from SLs [8]. In the same study, 3-epibartogenic acid and epigallocatechin, but not kaempferol glycoside, were shown to have α-amylase-inhibitory activities [8]. In other chemical characterization studies, three iridoids and eight polyphenols were identified in young SLs [4], and acteoside (a phenylethanol glucoside) and pedaliin (a flavonoid glucoside) were found as the major polyphenols [4,6,7]. One of the studies also reported that acteoside and isoacteoside had high antioxidant and anti-glycation activities compared to the other polyphenols identified, while the iridoids identified did not have such activities [4]. In a much earlier chemical study, pedaliin was shown to be present in SLs [9]. Regarding biological activities of SLs, gastro-protective, antimicrobial, radical-scavenging and anti-proliferative activities of the extracts were reported [10,11,12].

Cancer is a global health concern, and colorectal cancer is one of the most commonly diagnosed and leading causes of death among different cancers [13]. Carcinogenesis is a multi-step process involving the accumulation of multiple genetic alterations, the abnormal stimulation of growth, and the progression to a more malignant phenotype [14]. Oxidative stress, reflecting an imbalance between pro-oxidants and antioxidants and leading to persistent and excessive production of reactive oxygen species, is significantly implicated in the multi-step carcinogenesis at different stages [15,16]. Diet is regarded as an important modifier in the development of colorectal cancer; high intake of plant foods, such as vegetables, fruits, and whole grains, containing numerous bioactive phytochemicals has been related to lower risk of colorectal cancer [17]. Therefore, identification of plant food sources and their bioactive compounds targeting oxidative stress as well as critical events of the multi-step carcinogenesis is crucial for developing preventive and therapeutic strategy of colorectal cancers. 

Despite the several studies on SLs reported previously [4,5,6,7,8,9,10,11,12], there is little information available on the inhibitory activities of SL against cancers. Herein, we aimed to investigate in vitro antioxidant and anti-colon cancer activities of ethanol extract of SL (SLE). Major constituents of SLE contributing to the observed activities were also determined.

## 2. Materials and Methods

### 2.1. Materials

Cell culture media (RPMI and Macoy’s 5A) were purchased from Gibco (Rockville, MD, USA). Heat-inactivated fetal bovine serum (FBS) was purchased from Thermo Scientific (Logan, UT, USA). Penicillin and streptomycin were purchased from Welgene Inc. (Daegu, Korea). Multi-well plates and trans-well inserts were purchased from Corning Inc. (New York, NY, USA). Pedaliin was purchased from BioBioPha Co., Ltd. (Yunnan, China). All solvents (LC grade) were purchased from Fisher Scientific (Pittsburg, PA, USA). Unless indicated otherwise, all other materials and reagents were purchased from Sigma-Aldrich (St. Louis, MO, USA). 

### 2.2. Preparation of Sesame Leaf Extract

SL was obtained from a local farm (Cheongju, Korea) and was further washed, ground, and freeze-dried (PH1316, IshinBioBase, Yangju, Korea). Ten-fold volume of 70% ethanol was added to the dried SL, shacked vigorously using a voltex mixer for 6 h at room temperature, and centrifuged at 3500× *g* for 5 min (A320101, Gyrozen, Daejeon, Korea). The solvent of supernatant was removed using a speed vacuum (NB-503CIR, N-bioteck, Bucheon, Korea). The solid residue was regarded as an SLE, weighed for the calculation of extraction yield, and frozen at deep freezer (−70 °C) until further use.

### 2.3. Antioxidant Phytochemical Contents

Spectrophotometric analysis was conducted to determine total polyphenol, flavonoid, and carotenoid contents as described previously, with minor modifications [18,19,20,21]. 

Total polyphenol and flavonoid contents were determined by incubating SLE (reconstituted in ethanol, 500 μg/mL) with respective reagents and then reading the absorbance [18,19]. Briefly, for total polyphenol measurement, SLE was incubated with 7.5% sodium carbonate and Folin-Ciocalteu’s reagent in a ratio of 6:20:3 (*v*:*v*:*v*) for 30 min at room temperature. For total flavonoid measurement, SLE was incubated with 1 N sodium hydroxide and diethylene glycol in a ratio of 6:5:10 (*v*:*v*:*v*) for 1 h at room temperature. The absorbance of the resulting mixture was read at the wavelength of 650 nm (for total polyphenosl) or 415 nm (for total flavonoids) using a plate reader (Bio-Rad Laboratories, Hercules, CA, USA). Total polyphenol and flavonoid contents were calculated as mg gallic acid equivalent (GAE) per g of dried extract and mg quercetin equivalent (QE) per g of dried extract, respectively. 

Carotenoid content was determined by dissolving SLE in dimethyl sulfoxide (DMSO) and then reading the absorbance at 470 (A_470_), 647 (A_647_), and 663 nm (A_663_) in a plate reader (Bio-Rad Laboratories). The content was calculated by the equations of (1000A_470_ + 333.4837A_663_ − 1343.057A_647_) × 1.136 and is shown as mg per g of dried extract [20,21].

### 2.4. Antioxidant Activity Assay

2,2-Diphenyl-1-picrylhydrazyl (DPPH) radical scavenging activity and ferric reducing antioxidant power (FRAP) were assessed as described previously [22,23]. Briefly, SLE at concentrations of 50, 100, 250, and 500 μg/mL (reconstituted in ethanol) was mixed either with DPPH radical solution (1 mM in ethanol) in a 1:1 ratio (*v*:*v*) or with FRAP reagent in 2:3 ratio (*v*:*v*). In another experiment, pedaliin (at 0, 3.5, 7, 17.5 and 35 μM) was mixed with FRAP reagent in 2:3 ratio (*v*:*v*). The FRAP reagent was prepared by mixing 10 mM 2, 4, 5-tripyridyls-trazine in 40 mM hydrochloric acid, 20 mM ferric chloride, and 300 mM acetate buffer (pH 3.6) in a ratio of 1:1:10 (*v*:*v*:*v*). The reaction mixture was then incubated for 30 min at room temperature in the dark (in DPPH assay) or for 30 min at 37 °C (in FRAP assay). The resulting absorbance was read at 540 (in DPPH assay) or 595 nm (in FRAP assay) using a plate reader (Bio-Rad Laboratories). DPPH radical scavenging activity of SLE was expressed as a percentage by subtracting the absorbance of sample from absorbance of the control (DPPH radical solution mixed with ethanol only) and then dividing the value by the absorbance of control. FRAP of SLE and pedaliin was expressed as a percentage by dividing the absorbance of sample by absorbance of the control (ascorbic acid; 50, 100, 250, and 500 μg/mL in the experiment accessing FRAP of SLE; 35 μM in the experiment accessing FRAP of pedaliin).

### 2.5. Cell Culture and General Condition for Treatment

Human colon cancer cell lines, HT29 and HCT116 (Korean Cell Line Bank, Seoul, Korea), were maintained in RPMI and Macoy’s 5A media, respectively. Culture condition was 37 °C, 95% humidity, and 5% CO_2_. The media were supplemented with 10% FBS, 100 units/mL penicillin, and 0.1 mg/mL streptomycin. The stock solutions of SLE and pedaliin were prepared using DMSO and were further diluted to the desired final concentrations using the respective medium right before the treatment. The concentration of DMSO in the final condition did not exceed 0.2% (*v*/*v*), in all cell-based experiments.

### 2.6. Cell Viability Assay

HT29 and HCT116 cells (1 × 10^4^ cells/well) were seeded in 96-well plates. Cells were allowed to attach for 24 h and incubated with serial concentrations of SLE (0, 50, 100, 250, and 500 μg/mL) in FBS-free media for 24, 48, and 72 h. In another experiment, HCT116 cells (1 × 10^5^ cells/well) were incubated with serial concentrations of pedaliin (0, 3.5, 7, 17.5 and 35 μM) in FBS-free media for 72 h. After aspiration of medium, cells were incubated with 3-(4,5-dimethylthiazol-2-yl)-2,5-diphenyltetrazolium bromide (MTT) at 0.5 μg/mL for a further 4 h. DMSO was then added to solubilize the formazan precipitates, and the absorbance was read at 540 nm (Plate reader, Bio-Rad Laboratories, Hercules, CA, USA).

### 2.7. Flow Cytometry

HT29 and HCT116 cells (1 × 10^5^ cells/well) were seeded in 6-well plates. After starvation for 24 h in FBS-free media for cell cycle synchronization, cells were incubated with SLE at respective concentrations (0–500 μg/mL) in FBS-complete media for 72 h. Both floating and adherent cells were collected, washed with PBS, and spun down at 300× *g* for 3 min. The resulting pallet of cells was fixed with cold 70% methanol and then incubated with ribonuclease (1 μg/mL) and propidium iodide (50 μg/mL) for 30 min at 37 °C. Sub-G1, G1/G0, S, and G2/M cell populations were analyzed using a flow cytometer equipped with Cell Quest Pro software (BD Biosciences, Heidelberg, Germany). Cells at the number of 1 × 10^4^ were counted for each measurement.

### 2.8. Trans-Well Invasion and Migration Assay

HT29 and HCT116 cells (1 × 10^5^ cells/well), in FBS-free media containing SLE (0–100 μg/mL), were placed on trans-well inserts with an 8 μm filter. In another experiment, HCT116 cells (1 × 10^5^ cells/well) in FBS-free media containing pedaliin (0–7 μM) were placed on the inserts. The inserts were pre-coated with 20 μg of Matrigel for 2 h at 37 °C in the invasion assays, while intact inserts were used for the migration assays. After incubation for 24 (HT29 cells) or 48 h (HCT116 cells), the upper side of trans-well inserts were wiped out with a cotton swab. The cells penetrating through the insert toward the FBS-complete media-containing lower wells were stained with 0.1% crystal violet for 30 min. After fixation with methanol for 10 min and a subsequent wash with PBS, 1% sodium dodecyl sulfate was added to dissolve the stains, and the absorbance was read at 540 nm.

### 2.9. Scratch Would-Healing Migration Assay

HT29 and HCT116 cells, grown on 6-well plates (5 × 10^3^ cells/well), were scratched using a 2 mm-wide tip. The detached cells were removed by discarding the media. The remaining attached cells were incubated with FBS-free media containing SLE (0–100 µg/mL) and permitted to migrate for 24 (HT29) or 48 h (HCT116). The images of wound were captured in at least 7 different fields (iSolution Lite software, IMT i-solution, Burnarby, Canada), and the wound closure for the respective time was estimated (Image-J 1.x, NIH).

### 2.10. High-Performance Liquid Chromatography Anlaysis

Chromatographic analysis was carried out on an Agilent 1220 Infinity high-performance liquid chromatography (HPLC) system equipped with a UV/Vis detector (Agilent Technologies, Santa Clara, CA, USA) using an Eclipse plus C-18 column (5 μm, 4.6 × 150 mm, Agilent). The mobile phase comprised water containing 0.1% acetic acid (A) and acetonitrile containing 0.1% acetic acid (B), and flow rate was 0.7 mL/min. Gradient elution of the mobile phase was applied as follows: 0–5 min, 10% B; 5–15 min, 10–20% B; 15–25 min, 20–30% B; 25–35 min, 30–70% B; 35–40 min, 70–90% B; 40–50 min, 90%; 50–55 min, 90–10% B, 55–65 min, 10% B. The injection volume was 10 μL, and the detection wavelength was 340 nm. SLE and pedaliin standard were dissolved with methanol to prepare 10 and 1 mg/mL of stock solution, respectively, filtered using a 0.45 mm syringe membrane filter, and then diluted to a series of concentrations. Pedaliin in SLE was quantified by integrating the peak area of pedaliin and the calibration curve for pedaliin standard.

### 2.11. Data Analyses

All data were calculated as the mean ± SEM of at least triplicates. Differences between two groups were assessed by two-tailed Student *t*-test. Differences among multiple groups were assessed by one-way ANOVA and Duncan as a post hoc test. Dose and time dependence were assessed by regression analysis. A *p*-value lower than 0.05 was considered to be statistically significant.

## 3. Results and Discussion

### 3.1. Antioxidant Contents and Activities of SLE

Antioxidant phytochemicals are found ubiquitously in plant-based foods. We determined the content of major classes of antioxidant phytochemicals, such as polyphenols, flavonoids, and carotenoids, in SLE and, subsequently, their antioxidant activities. 

As shown in Table 1, SLE contained 36.5 mg GAE/g extract of total polyphenols, 129.2 mg QE/g extract of total flavonoids, and 537.7 mg/g extract of carotenoids. The total polyphenol and flavonoid contents of SLE were higher than those of ethanol extract of sesame seed (27.3 mg GAE/g dried extract and 11.37 QE/g dried extract, respectively) [24], which is consistent with previous reports on other plants showing that the leaves contain higher polyphenol contents than the seeds [25,26]. The total polyphenol and flavonoid contents of SLE found in our study were much higher than those of eight different leafy vegetables (80–317 µg ferulic acid equivalent/g and 31–151 µg rutin equivalent/g) [27], indicating that SLE is a rich source of antioxidant phytochemicals.

**Figure 1:**
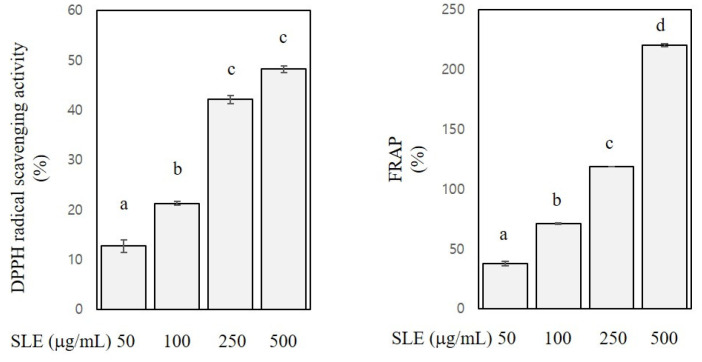
Antioxidant activities of SLE. 2,2-Diphenyl-1-picrylhydrazyl (DPPH) radical scavenging activities and ferric reducing antioxidant power (FRAP) of SLE at 50–500 μg/mL are expressed as a percentage. Significant differences among groups are marked using different letters (a–d) (*p* < 0.05).

DPPH assay and FRAP were performed to assess antioxidant activities of SLE. DPPH assay measures the activity of scavenging stable radicals [23], and FRAP measures the activity of reducing ferric ions to ferrous ions [22]. As shown in Figure 1, DPPH radical scavenging activity and FRAP of SLE at the concentration of 50–500 μg/mL ranged from 13 to 48% and from 38 to 221%, respectively. A radical scavenging activity of SLE was reported in a previous study [12], and our present results confirmed such activity. Further regression analysis revealed that the DPPH radical scavenging activity and FRAP were positively related with the SLE concentrations (R^2^ > 0.9, *p* < 0.001), indicating a dose-dependent antioxidant activity of SLE. Since polyphenols, flavonoids, and carotenoids contribute the most to the antioxidant properties of plant foods [28], the appreciable antioxidant activities of SLE found in our study are arguably ascribed to the high levels of these classes of phytochemicals in SLE. Our results corroborated the abundance of antioxidant phytochemicals in SLE and the corresponding antioxidant activities. Antioxidant phytochemicals are well known to exert beneficial properties against many chronic diseases, including cancers [28]. Therefore, we then further explored the anti-colon cancer properties of SLE using human cancer cell lines.

### 3.2. Growth-Inhibitory Activities of SLE in Human Colon Cancer Cells

Among several distinctive hallmarks of cancer cells exhibited in the multi-step carcinogenesis, the most fundamental one is to sustain promoted growth [14]. To examine anti-colon cancer potentials of SLE in vitro, we first determined the efficacy of SLE against the growth of colon cancer cells using two different human cancer cells lines, HT29 (colon adenocarcinoma cells) and HCT116 (colorectal carcinoma cells).

**Figure 2:**
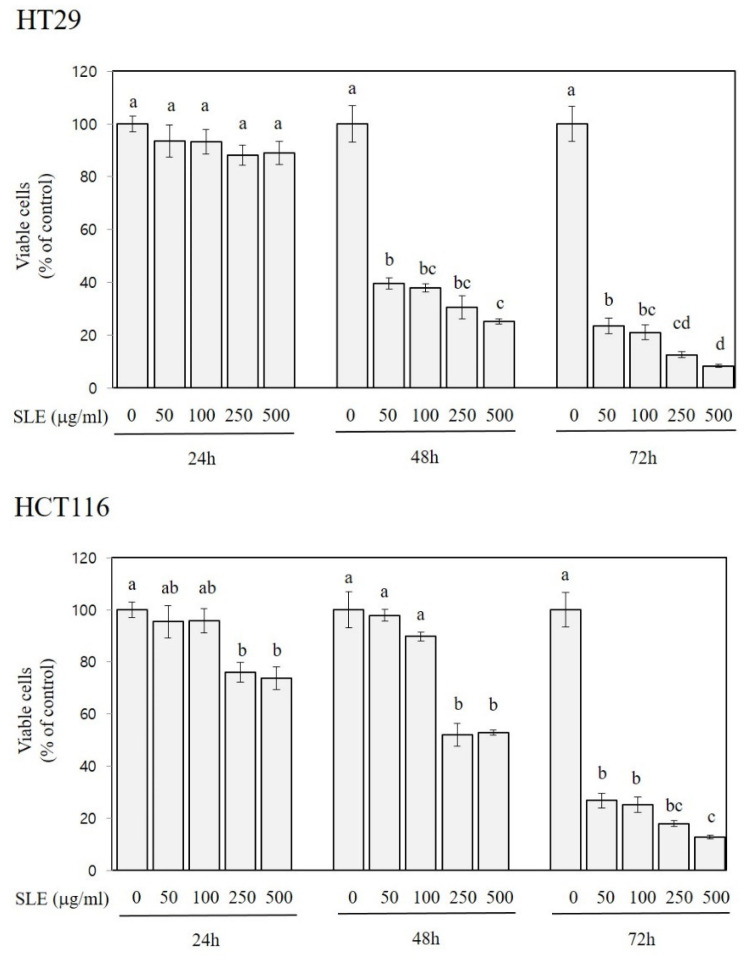
Growth-inhibitory activities of SLE in human colon cancer cells. Two different human colon cancer cell lines, HT29 and HCT116, were used for incubation with SLE at the concentrations of 0–500 μg/mL for indicated times (24, 48, and 72 h), and viable cells are expressed as a percentage of control. Significant differences among groups are marked using different letters (a–d) (*p* < 0.05).

As shown in Figure 2, the treatment of HT29 cells with SLE at the concentration of 50, 100, 250, and 500 µg/mL for 48 and 72 h decreased the growth by 60–75% and 77–92%, respectively. SLE was also efficacious in alleviating the growth of HCT116 cells; decreases in the growth by 24–26% (at 250 and 500 µg/mL), 47–48% (at 250 and 500 µg/mL), and 73–87% (at 50–500 µg/mL) were found at 24, 48, and 72 h time points, respectively. In both HT29 and HCT116 cells, the cell growth was inversely related to the treatment time or concentrations (R^2^ > 0.9, *p* < 0.001), indicating that the inhibitory activity of SLE against the cell growth was time- and dose-dependent. 

### 3.3. Apoptosis-Inducing and Cell Cycle-Arresting Activities of SLE in Human Colon Cancer Cells

Apoptosis is a vital process of programmed cell death for normal cell turnover, and cell cycle is an orderly sequence of events responsible for cell duplication and division. Defective apoptosis and dysregulated cell cycle progression are important factors that lead to abnormal growth of cancer cells [29]. To further characterize the growth inhibition by SLE, we examined the efficacy of SLE on apoptosis and cell cycle in HT29 and HCT116 cells by flow cytometry analysis. 

**Figure 3:**
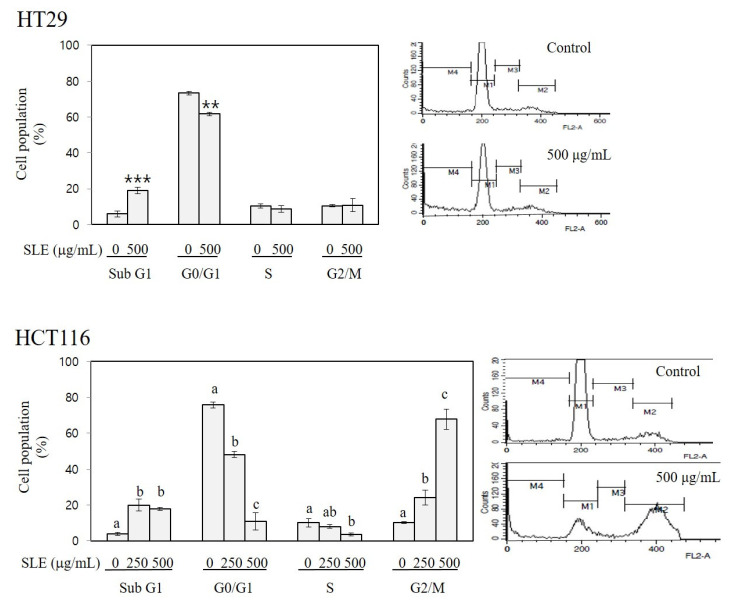
Apoptosis-inducing and cell cycle-arresting activities of SLE in human colon cancer cells. HT29 and HCT116 cells were incubated with SLE at the concentrations of 0–500 μg/mL for 72 h, and the population of cells in sub-G1 (M4, apoptotic), G0/G1 (M1), S (M3), and G2/M (M2) phases (%) were quantified by flow cytometry. Significant differences in comparison with the untreated control are marked using asterisks (** *p* < 0.01, *** *p* < 0.001). Significant differences among groups are marked using different letters (a–c) (*p* < 0.05).

As shown in Figure 3, treatment with SLE increased sub-G1 cell population to 3.2-fold of the control in HT29 cells (at 500 μg/mL) and to 4.7–5.2-fold of the control in HCT116 cells (at 250 and 500 μg/mL). Since the sub-G1 peak is indicative of cells incepting apoptosis [29], our results indicate an apoptosis-inducing activity of SLE. A prominent effect of SLE on cell cycle was found in HCT116 cells; treatment with SLE at 250 and 500 μg/mL increased the G2/M cell population to 2.3–6.6-fold of the control with a concomitant reduction in G0/G1 and S phase cell populations, indicating a G2/M arresting activity of SLE. These findings suggest that the growth inhibition by SLE (Figure 2) is mediated by the induction of apoptosis (in both HT29 and HCT116) and cell cycle arrest at G2/M (in HCT116). It is of importance to elucidate the detailed molecular mechanisms by which SLE induces apoptosis and G2/M cell cycle arrest in future investigations. 

### 3.4. Inhibitory Activities of SLE against Invasion and Migration in Human Colon Cancer Cells

Cancer metastasis is a series of complex pathological steps where proliferating neoplastic cells escape the primary tumor and form new colonies in secondary locations of the body. The main reason for cancer being a life-threatening disease is its metastatic properties. Invasion into surrounding stroma and migration through the extracellular matrix are inevitable steps that neoplastic cells must go through prior to penetrating blood and lymph vessels during metastatic cascade [14]. 

**Figure 4:**
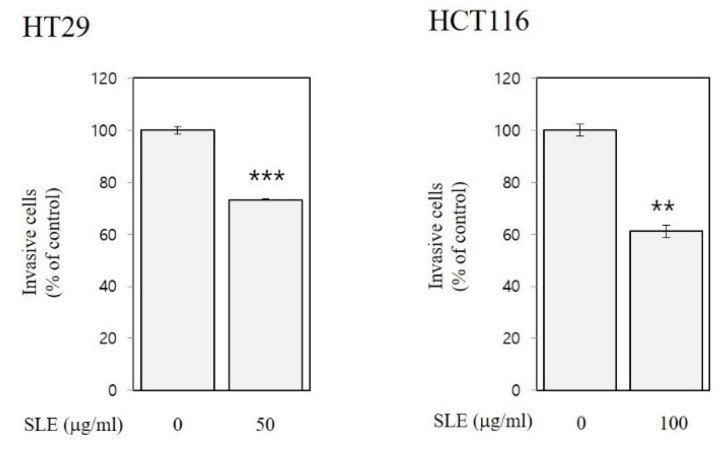
Inhibitory activities of SLE against invasion in human colon cancer cells. HT29 and HCT116 cells were incubated with SLE at an indicated concentration (0–100 μg/mL) for 24 and 48 h, respectively. Invasive cells are expressed as a percentage of control. Significant differences in comparison with the untreated control are marked using asterisks (** *p* < 0.01, *** *p* < 0.001).

**Figure 5:**
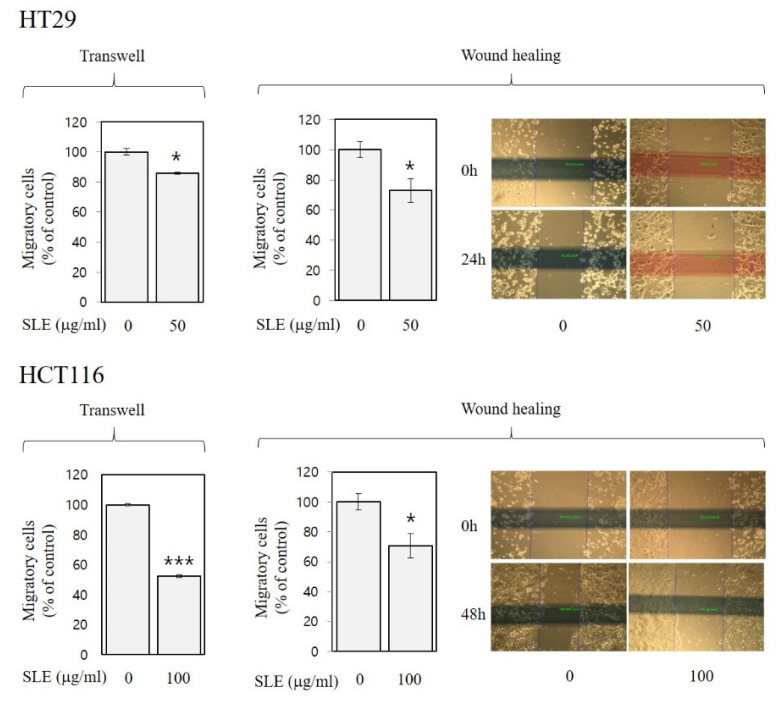
Inhibitory activities of SLE against migration in human colon cancer cells. HT29 and HCT116 cells were incubated with SLE at an indicated concentration (0–100 μg/mL) for 24 and 48 h, respectively, in two different migration assays (trans-well and wound-healing). Migratory cells are expressed as a percentage of control. Significant differences in comparison with the untreated control are marked using asterisks (* *p* < 0.05, *** *p* < 0.001).

As shown in Figure 4 and Figure 5, SLE alleviated invasion (by 14–39%) and migration (by 14–48%) of both HT29 and HCT116 cells, suggesting anti-metastatic activities of SLE in human colon cancer cells. Such inhibition against invasion (Figure 4) and migration (Figure 5) by SLE was found at non-cytotoxic conditions (≤100 μg/mL, 24 h time point), where SLE did not affect cell viability (Figure 2). Our results, therefore, suggest that the efficacy of SLE against invasion and migration is independent of the growth-inhibitory efficacy. Future studies on detailed molecular mechanisms for the inhibition of invasion and migration are warranted. 

We demonstrate in the present study that SLE exhibited antioxidant activities (Figure 1), suppressed the growth of human colon cancer cells possibly due to the induction of apoptosis and G2/M cell cycle arrest (Figure 2 and Figure 3), and attenuated metastatic events, such as invasion (Figure 4) and migration (Figure 5), in human colon cancer cells. To the best of our knowledge, this is the first report showing such activities of SLE systematically in a study. Plant extracts are a complex mixture of countless variety of compounds. It is still crucial to identify which components of SLE are most responsible for the observed activities. In this respect, we conducted chemical analyses next.

### 3.5. Pedaliin as a Major Constituent of SLE 

SL was reported to contain acteoside (a phenylethanol glucoside) and pedaliin (a flavonoid glucoside) as major constituents among the eight polyphenols identified [4,6,7]. Pedaliin was also noted to be present in SL in a much earlier study [9]. To examine if such polyphenols are also found in the SLE used in our study, HPLC analysis was performed. 

**Figure 6:**
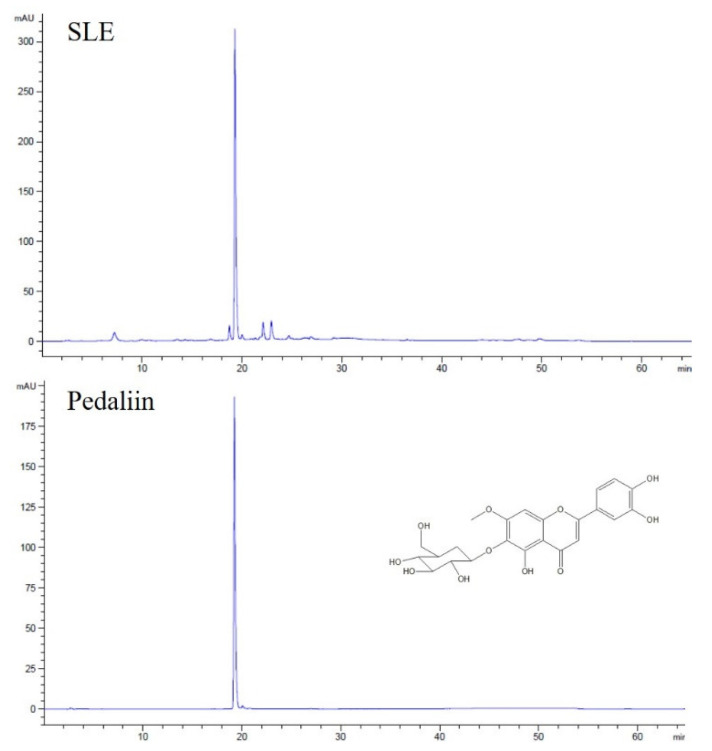
The HPLC chromatograms of SLE. Representative chromatogram of SLE and pedaliin standard are shown.

As shown in Figure 6, a single peak was predominant in the chromatogram of SLE at 340 nm. The peak was subsequently identified as pedaliin (6-hydroxylueolin 7-methyl ether 6-glucoside; pedalitin-6-*O*-glucoside) based on the retention time of pedaliin standard in the HPLC chromatogram (Figure 6) and mass spectroscopic data (data not shown). For further quantitation, the analytic method was validated in terms of linearity and sensitivity. As shown in Table 2, a calibration curve was constructed with a correlation coefficient (R^2^) greater than 0.99 in the range of 1–500 μg/mL of pedaliin. The limit of detection (LOD) and limit of quantitation (LOQ) were obtained at 0.040 and 0.135 μg/mL, respectively. Pedaliin in SLE was then quantified in triplicates, and the content was found to be 32.7 ± 0.16 mg/g extract. Such quantity corresponds to approximately 0.36% (*w*/*w*) in dried SL, which was in the range reported previously (0.01–2.05% in dried SL) [4]. Acteoside, reported as another major constituent of SL [4,6,7], was not detected in SLE used for our study under our experimental conditions. Differences in genotypes within a plant species, cultivation condition, timing, and region, and experimental conditions may result in a significant variation in the profile and quantity of phytochemicals measured [30,31]. Our results indicated that pedaliin is a main polyphenol of SLE, which led us to further determine whether pedaliin is a bioactive component conferring antioxidant (Figure 1) and anti-colon cancer activities (Figure 2, Figure 3, Figure 4 and Figure 5) to SLE.

### 3.6. FRAP and In Vitro Anti-Colon Cancer Activities of Pedaliin 

Previously, pedaliin was reported to exhibit considerable DPPH radical scavenging activity (2.0 mmol Trolox equivalent/g) and oxygen radical absorbance capacity (24.4 mmol Trolox equivalent/g) [4]. However, no other biological activities of pedalliin than the antioxidant activities have been reported. To confirm the antioxidant activity of pedaliin and to examine its in vitro anti-colon cancer activities, FRAP, cell viability, and trans-well assays were performed. The selected concentrations of pedaliin were 3.5, 7, 17.5, and 35 µM since these were estimated to correspond to 50, 100, 250, and 500 µg/mL of SLE, respectively, based on the content of pedaliin in SLE (32.7 mg/g extract). 

**Figure 7:**
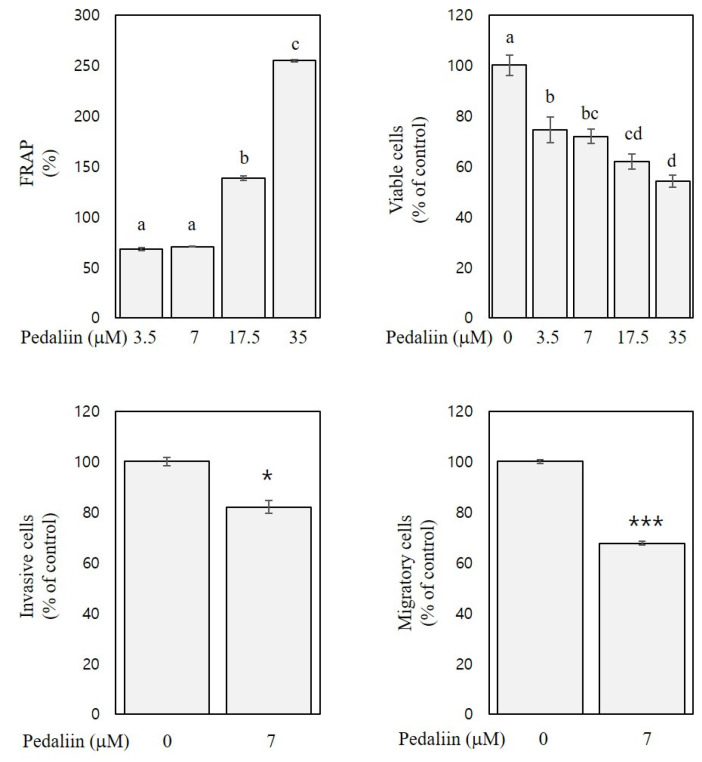
FRAP and in vitro anti-colon cancer activities of pedaliin. FRAP and inhibitory activities of pedaliin against growth (at 72 h), invasion (at 48 h), and migration (at 48 h) of HCT116 cells are expressed as a percentage of the control. Significant differences among groups are marked using different letters (a–d) (*p* < 0.05). Significant differences in comparison with the untreated control are marked using asterisks (* *p* < 0.05, *** *p* < 0.001).

As shown in Figure 7, FRAP of pedaliin at the concentration of 3.5–35 μM was 68–254% and dose-dependent (R^2^ > 0.9, *p* < 0.001), indicating the substantial antioxidant activity of pedaliin. The FRAP of pedaliin (Figure 7) was comparable to those of SLE (Figure 1, right panel), which suggests that pedallin significantly contributes to the antioxidant activities of SLE. Moreover, the cell viability (at 72 h) and trans-well assays (at 48 h) with HCT116 cells revealed that pedaliin inhibited growth (by 24–45%, at the concentrations of 3.5–35 µM), invasion (by 18%, at the concentration of 7 µM), and migration (by 33%, at the concentration of 7 µM) (Figure 7). These results suggest the growth-inhibitory, anti-invasive, and anti-migratory activities of pedaliin in human colon cancer cells. To the best of our knowledge, this is the first report showing in vitro anti-colon cancer activities of pedaliin. It is noteworthy that the activities of pedaliin against growth, invasion, and migration of HCT116 cells (Figure 7), however, did not achieve the full extent of corresponding activities of SLE found in HCT116 cells (Figure 2, lower panel, 72 h; Figure 4, right panel; and Figure 5, lower left panel). Plant extracts have frequently shown to exhibit more potent biological activities than an isolated single compound present in the extract [32]. Our results suggest that pedaliin is a compound that makes a considerable, but still partial, contribution to anti-colon cancer activities of SLE found in our study. It can be speculated that pedaliin may interact with other phytochemicals that were beyond the range and scope of the analysis performed in the current study but that are present in SLE, which interaction might produce an additive and/or synergistic effect. Further studies addressing this issue are needed. 

Taken together, we demonstrated that SLE possesses in vitro antioxidant and anti-colon cancer activities and that pedaliin is a major constituent contributing such activities. These findings are expected to provide a scientific basis for development and application of functional foods and nutraceuticals using SLE and pedaliin. Further research is needed to standardize a pedaliin-enriched SLE, to determine whether the activities of SLE and pedaliin found in our study are reproduced in animals and finally humans, and to elucidate the underlying molecular mechanisms of the inhibitory action.

## 4. Conclusions

SLE is a rich source of antioxidant phytochemicals, such as total polyphenols, total flavonoids, and carotenoids, and correspondingly exhibited considerable antioxidant activities, such as radical scavenging activity and FRAP. SLE inhibited the growth of HT29 and HCT116 human colon cancer cells, which was due to the induction of apoptosis (both in HT29 and HCT116) and G2/M cell cycle arrest (HCT116 cells). SLE was also efficacious in alleviating invasion and migration of HT29 and HCT116 cells in non-cytotoxic conditions. Pedaliin was present in the SLE used in our study as a major polyphenol and showed significant FRAP, growth-inhibitory, anti-invasive, and anti-migratory activities. This study demonstrated that SLE possesses in vitro antioxidant and anti-colon cancer activities and that pedaliin is a major constituent contributing such activities. Our findings provide basic information for further mechanistic, animal, and ultimately translational studies to develop SLE and pedaliin as effective preventive and therapeutic agents against human colorectal cancers and other diseases.

## Figures and Tables

**Table 1:** Antioxidant contents of sesame leaf extract (SLE).

Phytochemicals	Contents
Extraction yield	11.2 ^1^
Total polyphenols	36.5 ± 1.1 ^2,3^
Total flavonoids	129.2 ± 20.9 ^2,4^
Carotenoids	537.7 ± 67.6 ^2,5^

^1^ % (*w*/*w*). ^2^ Mean ± SEM of ≥3 determinations. ^3^ mg gallic acid equivalent/g dried extract. ^4^ mg quercetin equivalent/g dried extract. ^5^ mg/g dried extract.

**Table 2:** Regression data, limit of detection, and limit of quantification for pedaliin.

Concentration Range Tested (μg/mL)	Linear Regression Data		LOD ^1^ (μg/mL)	LOQ ^1^ (μg/mL)
	Calibration Curve ^2^	Correlation Coefficient (R^2^)		
1–500	Y = 38.919X + 98.892	0.9997	0.040	0.135

^1^ LOD: limit of detection; LOQ: limit of quantification. ^2^ Y: peak area; X: concentration of pedaliin.

## Data Availability

The datasets generated for this study are available on request to the corresponding author.
